# Utilizing vancomycin as secondary prophylaxis for the prevention of recurrent *Clostridioides difficile* infection

**DOI:** 10.1017/ash.2023.215

**Published:** 2023-09-29

**Authors:** Zeitler Kristen, Andrew Nguyen, Candice Mateja, Ripal Jariwala, Carlos Bertran-Rodriguez, Cynthia Mayer, Diep Nguyen, Mindy Sampson

## Abstract

**Background:** Recurrent *Clostridioides difficile* infection (CDI) is associated with significant morbidity, mortality, and healthcare-related costs. Although data are minimal, agents including oral vancomycin have been used as secondary prophylaxis to prevent recurrent CDI. **Methods:** We conducted a randomized, double-blind, placebo-controlled trial to determine the effectiveness of vancomycin at preventing CDI from October 2019 to September 2022. Eligible patients had a history of at least 1 episode of CDI and were receiving systemic antibiotics for another condition. Participants were randomized 1:1 to oral vancomycin 125 mg by mouth twice daily and were interviewed at 1, 2, and 3 months thereafter to assess recurrence. We enrolled 26 patients: 15 completed the 1-month interview, 12 completed the 2-month interview, and 11 completed the full study. Those 15 participants who did not complete the 3-month interview were considered dropouts. The final sample for this study included those 11 participants who completed all interviews. Demographics and outcomes are shown in Table 1. **Results:** One case of recurrent CDI was reported at the 1-month interview and a second was reported at 3 months; both cases had received the placebo. The study was terminated early due to low enrollment. **Conclusions:** Although our results did not reach statistical significance and this study was limited in small sample size, our findings suggest that secondary prophylaxis with oral vancomycin may be beneficial in patients who are actively receiving antibiotics, which is consistent with prior retrospective studies. Future studies with larger sample sizes are still needed to examine this important question of whether secondary prophylaxis is useful for preventing recurrent CDI.

**Figure:**
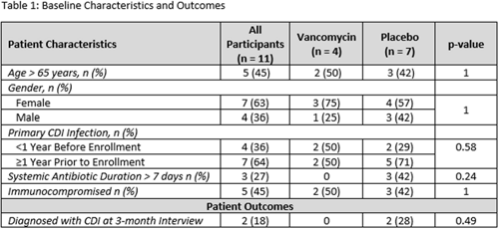

**Disclosure:** None

